# Diacetyl and Other Ketones in e-Cigarette Aerosols: Some Important Sources and Contributing Factors

**DOI:** 10.3389/fchem.2021.742538

**Published:** 2021-09-23

**Authors:** Kevin McAdam, Gareth Waters, Serban Moldoveanu, Jennifer Margham, Anthony Cunningham, Carl Vas, Andrew Porter, Helena Digard

**Affiliations:** ^1^ McAdam Scientific Ltd., Eastleigh, United Kingdom; ^2^ Research and Development, British American Tobacco, Southampton, United Kingdom; ^3^ R.J. Reynolds, Winston-Salem, NC, United States; ^4^ Longwell Green, Bristol, United Kingdom; ^5^ Montreal, QC, Canada

**Keywords:** e-cigarette, diacetyl, flavors, acetyl propionyl, acetoin, pyrolysis - gas chromatography

## Abstract

**Background:** Concerns over the presence of the diketones 2,4 butanedione (DA) and 2,3 pentanedione (AP) in e-cigarettes arise from their potential to cause respiratory diseases. Their presence in e-liquids is a primary source, but they may potentially be generated by glycerol (VG) and propylene glycol (PG) when heated to produce aerosols. Factors leading to the presence of AP, DA and acetoin (AC) in e-cigarette aerosols were investigated. We quantified direct transfer from e-liquids, examined thermal degradation of major e-liquid constituents VG, PG and 1,3 propanediol (1,3 PD) and the potential for AC, AP and DA production from sugars and flavor additives when heated in e-cigarettes.

**Method:** Transfers of AC, AP and DA from e-liquids to e-cigarette aerosols were quantified by comparing aerosol concentrations to e-liquid concentrations. Thermal generation from VG, PG or 1,3 PD e-liquids was investigated by measuring AC, AP and DA emissions as a function of temperature in an e-cigarette. Thermal generation of AC, AP and DA from sugars was examined by aerosolising e-liquids containing sucrose, fructose or glucose in an e-cigarette. Pyrolytic formation of AP and DA from a range of common flavors was assessed using flash pyrolysis techniques.

**Results:** AC transfer efficiency was >90%, while AP and DA were transferred less efficiently (65%) indicating losses during aerosolisation. Quantifiable levels of DA were generated from VG and PG, and to a lesser extent 1,3 PD at coil temperatures >300°C. Above 350°C AP was generated from VG and 1,3 PD but not PG. AC was not generated from major constituents, although low levels were generated by thermal reduction of DA. Aerosols from e-liquids containing sucrose contained quantifiable (>6 ng/puff) levels of DA at all sucrose concentrations tested, with DA emissions increasing with increasing device power and concentration. 1% glucose, fructose or sucrose e-liquids gave comparable DA emissions. Furanose ring compounds also generate DA and AP when heated to 250°C.

**Conclusions:** In addition to less than quantitative direct transfer from the e-liquid, DA and AP can be present in the e-cigarette aerosol due to thermal decomposition reactions of glycols, sugars and furanonse ring flavors under e-cigarette operating conditions.

## Introduction

Electronic nicotine delivery devices (ENDS), or e-cigarettes, have the potential for being less harmful alternatives to conventional combustion cigarettes (Shahab et al., 2017). They operate by heating e-liquids to produce an inhalable aerosol on puffing. E-liquids are composed of aerosol-formers (usually propane-1,2,3-triol or “vegetable” glycerol (VG), and/or propane-1,2-diol or propylene glycol (PG) and much less frequently 1,3-propylene diol (1,3-PD)), a viscosity regulator (water), nicotine and flavorings. When activated, the heating coil (or coils) used to generate the aerosol reaches temperatures in normal operation of between 145°C and 330°C, depending on the power supplied (Chen et al., 2018).

**FIGURE 1:**
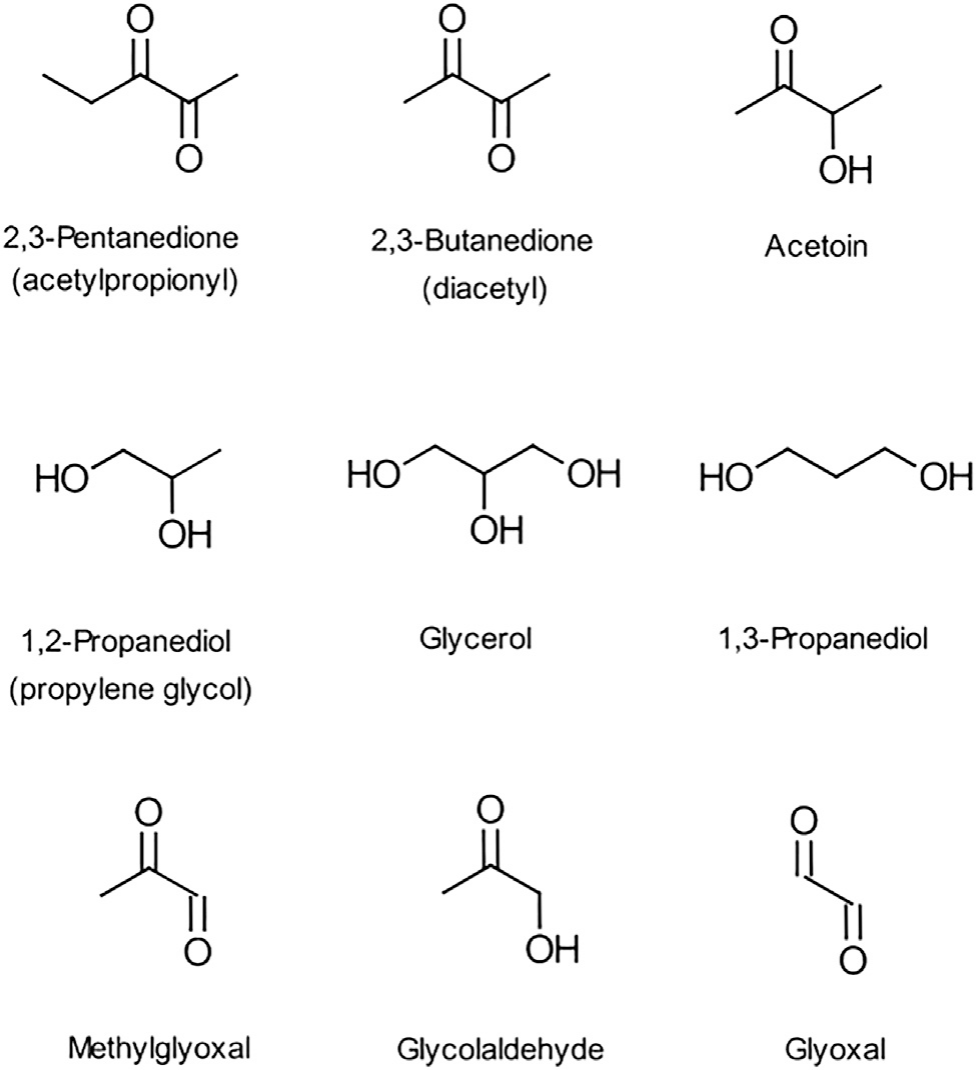
Structures of the ketones, glycols, methylglyoxal, glycolaldehyde and glyoxal.

There are concerns about the use of e-liquid ingredients that may introduce unintended health risks to the consumer. One ingredient of particular concern is the flavor compound, diacetyl (2,3-butanedione, DA), which is a volatile α-diketone with the structure shown in Figure 1. It imparts a buttery/vanilla flavor and occurs naturally in a variety of foodstuffs such as dairy products, beer, coffee, honey and fruits (Clark and Winter, 2015). DA is also used widely in foods as a flavor additive and is “generally recognised as safe” (GRAS) when used for this purpose. However, there is strong evidence, from both occupational exposure and animal studies, that inhalation of high levels of DA vapour can cause serious lung damage in humans (NIOSH, 2016). Compounds with flavors similar to DA (Figure 1) such as acetyl propionyl (2,3-pentanedione, AP), an α-diketone homolog of diacetyl, and acetoin (AC), a hydroxyl ketone, are also used in foodstuffs. Acetyl propionyl has also been shown to cause lung damage in exposed animals, while in contrast, AC does not have the reactive α-dicarbonyl group and is thought to be considerably less hazardous than DA (NIOSH, 2016). In 2016 the National Institute for Occupational Safety and Health (NIOSH) established recommended exposure limits (RELs) for DA and AP but not for AC.

As early as 2008 there were health concerns amongst vapers about the use of DA as a flavorant in e-liquids (Farsalinos et al., 2015; Vas et al., 2019). However, over the last decade a growing number of surveys have continued to identify the presence of DA, AC and AP in American, Canadian and European e-liquids (Farsalinos et al., 2015; Barhdadi et al., 2017; Moldoveanu et al., 2017; LeBouf et al., 2018; Vas et al., 2019; Czoli et al., 2019), (Supplementary Table S1), and aerosol emissions from commercial e-cigarettes (Allen et al., 2016; Margham et al., 2016; Sleiman et al., 2016; Klager et al., 2017; Moldoveanu et al., 2017; Melvin et al., 2020).

Given the volatile nature of these compounds it can be anticipated that they would volatilise and transfer from the e-liquid to the aerosol during puffing. However, despite the growing range of studies identifying these compounds in e-liquids or e-cigarette emissions, surprisingly no study has clearly evaluated emissions from e-cigarettes containing known e-liquid content at levels relevant to commercial e-liquids. The closest reported study was that of Farsalinos et al. (2015), who created three experimental e-liquids at very high DA and AP contents, and identified near-quantitative transfer to the aerosol even though the concentrations were significantly higher than measured in the great majority of commercial e-liquids. Moldoveanu et al. (2017) also measured both e-liquid and aerosol DA concentrations in their study. Both of these studies examined aerosol emissions from freshly prepared e-liquids. However, Vas et al. (2019) demonstrated that DA and particularly AP are chemically reactive in e-liquids, generating a range of reaction products over a period of weeks after e-liquid manufacture. It can be hypothesised therefore that the operation of such chemical reactions during product shelf-life might influence the efficiency with which these species are transferred from e-liquid to aerosol during puffing. Consistent with this, Pankow et al. (2018) in a study of gas/particle partitioning of e-cigarette flavors, commented on the formation of significant amounts of reaction products from DA. Understanding of the hazards associated with DA and AP exposure during vaping, particularly dosimetry aspects, would therefore be advanced by insight into the efficacy with which these species transfer to the aerosol during vaping.

In addition, there are indications of other sources of these compounds in e-cigarette aerosols. For example, we have recently shown (Vas et al., 2019) that when AC is added to e-liquids, some of it is gradually oxidised to DA during storage at room temperature. Oxidation of AC is accelerated by higher pH conditions such as those obtained in nicotine-containing solutions. Thus, vapers were at risk of DA exposure without DA being initially present in the e-liquid formulation. Our findings were consistent with the relative concentrations of DA and AC measured in commercial e-cigarette aerosols by Allen et al. (2016) for all but one of the 51 e-liquids they analysed.

It is also plausible that these compounds may arise in e-cigarette aerosols from thermal degradation sources. At the higher temperatures experienced in e-cigarettes it has been clearly established (Uchiyama et al., 2020) that the aerosol formers - VG and PG - can undergo thermal degradation to a number of lower molecular weight carbonyls such as acrolein and formaldehyde. There is indirect evidence from gas phase catalytic dehydration of glycerol that DA can be formed via an addition reaction at temperatures of about 300°C. Consistent with this, two studies (Behar et al., 2016; Sleiman et al., 2016) have identified the presence of DA in the aerosol from e-cigarettes containing e-liquids free from DA, including neat PG and neat VG. Recent model studies using a microwave reactor heated to 180°C for several minutes have provided additional evidence for AP and DA production from VG and PG (Melvin et al., 2020). Together, these observations strongly suggest that thermal degradation of the main aerosol formers can produce DA, AC or AP during e-cigarette use. However, further information is needed on this possibility, particularly the threshold temperatures for ketone formation, the extent with which the ketones are generated, and the relative efficiency of generation by different aerosol former compounds.

Compounds besides PG and VG, such as flavors, may also potentially degrade thermally during aerosolization leading to the formation of DA, AC or AP in e-cigarette aerosols. One such class of additive, saccharides, have been used to create sweet flavored e-liquids (National Academies of Sciences Engineering, and Medicine, 2018), although prohibited under voluntary regulations in some jurisdictions (AFNOR 2016). Two studies (Kubica et al., 2014; Fagan et al., 2017) have shown both that a high proportion of e-liquids contain sucrose and quantified its presence in those e-liquids. In addition to intentional addition (Vape Club, 2020), sugars can also be introduced as natural components of flavor additives such as fruit extracts (Myeliquid, 2021; Soussy et al., 2016; and Fagan et al., 2017) demonstrated the operation of thermal breakdown reactions of sucrose, glucose and sorbitol under vaping conditions. We had concerns that DA or AP may also be formed from sugars during vaping since DA formation has been observed during caramelisation of sucrose under non-vaping conditions (Monte and Maga 1981). Finally, a range of more volatile flavors were also investigated for their potential to form DA, AC and AP on heating in e-cigarettes.

The current paper therefore describes our investigations into potential sources of DA, AP and AC in e-cigarette aerosols. The paper reports results from four sets of experiments. The first was a study of the transfer efficiencies of DA, AP and AC from e-liquids to the e-cigarette aerosol during typical e-cigarette shelf-life times. The second experiment involved the analysis of aerosols generated at different power levels from model e-liquids containing only either PG, VG or 1,3 PD, plus sufficient water to ensure compatibility with the wicking characteristics of the e-cigarette device. The third series describes the analysis of aerosols from e-liquids containing sucrose concentrations in the range of 0–10%, as well as from 1% solutions of glucose and fructose. The final experiments were a pyrolysis screening exercise examining production of DA and AP from a range of common flavor compounds.

## Methods

### Reagents

Pharmaceutical grade glycerol (99.9% purity) was obtained from Sigma Aldrich (Gillingham, United Kingdom. Product code 49779, lot number BCBQ6768V); pharmaceutical-grade propylene glycol (>99% purity) was obtained from Sigma Aldrich, Fluka (code 82281, lot number BCBQ0147V) and pharmaceutical-grade nicotine (99.4% purity by non-aqueous titrimetric determination) was obtained from Siegfried (Minden, Germany. Lot number 1517/024). The water used in the study was city water connected to a Millipore (Watford, United Kingdom) deionised ultra-filter (DIUF) and purified to a water resistivity value of 18.2 MΩ.cm at 25°C. 10 M sodium hydroxide solution was obtained from Sigma Aldrich. Acetoin was sourced from Sigma Aldrich (product code: A17951, lot number MKBQ2240V), with a declared purity of 99.3% by GC. Diacetyl (a mix of the monomer and dimer) was sourced from Sigma Aldrich (product Code: B85307, lot number BCBM5232V), with a declared purity of 97%. Acetyl propionyl was sourced from Sigma Aldrich (product Code 241962, lot number MKBB7504V), with a declared purity of 97.1%. Sucrose, glucose, and fructose were supplied by Sigma Aldrich. Compounds for the pyrolysis study were variously obtained from Sigma-Aldrich (St. Louis, MO, United States), Vigon International (East Stroudsburg, PA, USA), Tobacco Technology Inc. (Eldersburg, MD, USA), I.P. Callison and Sons (Lacey, WA, USA), Vantage Oleochemicals (Chicago, IL, USA), and Archer Daniels (Chicago, IL, USA).

### AC, AP and DA Transfer Studies

An e-liquid formulation (2,500 ml) consisting of glycerol (48.76% w/w), 1,2 propylene glycol (25% w/w), water (25% w/w) and nicotine (1.24% w/w) was prepared at British American Tobacco’s, (BAT) R&D laboratories. 1,3-PD was not used in this formulation due to its infrequent use in commercial e-liquids compared to PG and VG. The formulation was sent to Enthalpy Analytical (Durham, NC, United States), where it was split into four e-liquid sub-samples. The first sub-sample was untreated and acted as a control. The second sub-sample was spiked with 1,000 μg/ml AP, the third was spiked with 1,000 μg/ml of AC and the fourth was spiked with 1,000 μg/ml of DA.

The four sub-samples were stored in clear volatile organic analyte vials at 20 ± 2°C and 60% RH for time periods up to 64 days, before being analysed in triplicate. Not all analyses were conducted at each time-point for each e-liquid, as reported below. The use of a time-course approach allowed us to examine the transfer of these species during a typical shelf-life period, as well as at time of e-liquid manufacture. Also, because concentrations of DA, AP and AC decline considerably in e-liquids over a 64 day period (Vas et al., 2019) the experimental design allowed for transfer efficiency to be examined across a range of compound concentrations that were consistent with previously reported ketone emissions from e-cigarettes (Farsalinos et al., 2015, Supplementary Table S1). The sub-samples were analysed in triplicate for AC (LOD: 2 μg/ml, LOQ: 20 μg/ml), AP (LOD: 1 μg/ml, LOQ: 10 μg/ml) and DA (LOD: 1 μg/ml, LOQ: 10 μg/ml) by GC/MS in accordance with Enthalpy SOP ENT-225.

In these experiments, aerosol testing was also conducted by Enthalpy Analytical (Durham, NC, United States). Using the same schedule as for the e-liquid analyses the aerosol emissions were analysed for AC, AP and DA, with three replicates at each time point. Aerosol and e-liquid concentrations were both determined at the same point and analysed as part of the same analytical batch to minimise interference from time-based reactions of the investigated species (Vas et al., 2019). Aerosols of the e-liquids were generated using a Vype eTank clearomiser. Two ml of the e-liquid samples were placed inside the tank and left to “wick” for 5 minutes. The tank, battery and mouthpiece were assembled. The device was operated at an angle of 45° (battery side down) and the voltage was set at 3.8 V. Aerosols were collected for 100 puffs with a puff volume of 80 ml, a puff duration of 3 s, a puff interval of 30 s and a square wave puff profile. The device button was turned on manually 1 s prior to each puff. Device weight loss was recorded as a measure of aerosol mass generated during puffing. Methods used by Enthalpy Analytical for the analyses of AC, AP and DA have previously been reported by Vas et al. (2019).

Expected aerosol per puff yields of the analytes (assuming 100% transfer from e-liquid to aerosol) were calculated from the e-liquid concentrations of the analytes and the mass loss per puff as follows:

$$Expected\;yield\;\left(µg/puff\right)=\;\left(W\times C\right)/\left(10^{3}\;\times \;D\right)$$

Where *W* is the weight loss of the e-cigarette per puff (mg), *D* is the density of the e-liquid (g/ml) and *C* is the e-liquid concentration of the component (µg/ml). The e-liquid density, *D*, was calculated as 1.123 g/ml from the densities of the individual components and the proportions of the un-spiked e-liquid components (PubChem 2018).

The transfer efficiency of these compounds from the e-liquid to the aerosol was estimated by comparing the expected and measured aerosol yields on a percentage basis. The expected yields were based on the total weight of e-liquid lost during aerosol generation and the concentration of the component in the e-liquid. Thus:

$$Transfer\;Efficiency\;\left(\%\right)=\frac{Y\times D\times 10^{5}}{W\times C}$$

where: *Y* = measured aerosol yield of the component (µg/puff).

### Thermal Generation of AC, AP and DA by e-Liquid Solutions of VG, PG and 1,3-PD

The potential for major e-liquid components to generate DA, AP and AC was tested by creating three model e-liquid formulations comprising only one of the aerosol formers, plus a level of water appropriate to ensure a suitable viscosity to operate effectively with the test e-cigarette. These model e-liquid formulations were heated (separately) in an atomiser, with operating power levels increased systematically from 10 to 35 W in order to create a range of temperatures in the atomiser covering normal e-cigarette operating temperatures as well as the higher temperatures that might be encountered in dry wicking or over-powered e-cigarette scenarios. Parameters for the study were defined by published data on the generation of thermal decomposition products from e-liquids where power levels up to 85 W have been applied to an e-cigarette (Uchiyama et al., 2020), as well as reported e-cigarette operating temperatures. Schripp et al. (2013) measured heating coil temperatures of around 350°C, Geiss et al. (2016) reported temperatures >300°C, (Zhao et al., 2016) reported coil temperatures up to 300°C, (Chen et al., 2018) reported coil temperatures of 110–185°C operating with a PG e-liquid under fully wet conditions, 145–334°C with a wet-through-wick condition, and 322–1,008°C under dry wick conditions. In the present study, screening experiments showed e-cigarette power levels up to 35 W could generate the coil temperatures described above.

The model e-liquid formulations were prepared at British American Tobacco (Southampton, United Kingdom) and comprised (on a % w/w basis) a) 75% VG + 25% water, b) 91% PG + 9% water and c) 91% 1,3-PD + 9% water. While these % water levels are seen in some commercial e-cigarette e-liquids, their inclusion in this experiment was driven primarily by the need to ensure effective wicking of the e-liquids with the e-cigarette used.

Commercially available modular e-cigarettes were used for the generation of the emissions. The e-cigarette comprised an Aspire Nautilus mini 2 ml tank, an Aspire 1.8 Ω BVC atomiser with a cotton wick and a bottom vertical coil (Aspire 2016). For powers up to 30 W, a 30 W eLeaf iStick battery/power supply was used. This has a 2,200 mAh battery with variable voltage (2.0–8.0V) and power (5–30 W) settings (Eleaf 2016). For powers greater than 30 W a 40 W eLeaf iStick battery/power supply was used.

Analytical testing, test-piece assembly, machine puffing, and aerosol collection were conducted by Labstat International (Kitchener, Ontario, Canada). The Aspire tank was pre-filled with 2 ml of test e-liquid and allowed to “wick” for 5 minutes. The filled tanks were then connected to fully charged batteries. Aerosols were generated with a puffing regime of 80 ml puff volume, 3 s puff duration and 30 s interval. Five replicate collections of 25 puffs were obtained at power settings of 10, 12, 14, 16, 18, 20, 25, 26.5, 27.5, 28.5, 30, 32 and 35 W for each of the three e-liquids. Three replicate air blanks were also obtained. The device was weighed before and after aerosol collection to record the device mass loss, which is a measure of the amount of aerosol generated.

The coil temperatures operating in the Aspire device during puffing were determined using a RS Pro Type K thermocouple (RS Number: 131–4,749) that had a calibrated temperature range of 0°C to +700°C. The thermocouple was attached to the atomiser wicking material as close as possible to, but not touching, the coil using thermal insulation tape. A second RS Pro Type K thermocouple (as a control) was used to monitor the ambient air temperatures of the laboratory. Temperatures were recorded using a thermocouple data logger and software, manufactured by Pico Technology Limited. Temperatures were measured under identical conditions to the chemical analyses described above. For temperature measurements conducted at different power settings, a fresh atomiser was used for each power level, with the tank filled to 2 ml before temperature measurements commenced. A Borgwaldt A14 single port smoke machine engine was used with identical puffing conditions to the aerosol measurements conducted by Labstat; temperature measurements were conducted for all 25 puffs.

Labstat method TMS-00155 (Carbonyls and Dicarbonyls) was used for analysis of aerosol carbonyl and dicarbonyl compounds in these studies (Bao 2015). Aerosols and blanks were collected using a 44 mm Cambridge filter pad (CFP) and a cryogenic impinger containing 20 ml of acetonitrile at −35°C. The CFP was extracted using the same impinger solution. The carbonyls captured in the impinger solution were derivatised using pentafluorobenzyl-hydroxylamine (PFBHA) prior to analysis by gas chromatography mass spectrometry (GC-MS). The column used was a Rtx-5ms (30 m × 0.25 mm × 0.25 µm) with an injector volume of 1 µl and a flow of 1 ml/min. The injector temperature was 260°C with an oven temperature regime of 70°C for 30 min, followed by 5°C/min to 220°C and then 30°C/min to 280°C and hold for 2 min. The mass spectrometer (MS) transfer temperature was 260°C, MS source temperature 250°C and MS quad temperature 150°C using a SIM scan mode. LOD and LOQ values for these analyses are provided in Supplementary Table S2.

### Generation of AC, AP and DA From e-Liquids Containing Sugars

The investigation of sugars as potential sources of AC, AP and DA in e-cigarettes was carried out in two parts. The first part focused on sucrose at concentrations of up to 1% in the e-liquids, to reflect levels potentially present in e-liquids. Also included were e-liquid formulations containing 1% fructose and 1% glucose to determine if the monosaccharides also produced the aerosol ketones to the same extent as sucrose. In these formulations, increasing sugar levels were incorporated by reducing water content. A second stage of this experiment involved analysis of aerosols from e-liquids with higher sucrose levels (up to 10%, assembled by increasing sucrose and reducing glycerol) in order to compare the efficiency of diketone production from sucrose and VG, and to provide greater clarity on trends in AP emissions.

E-liquids used in the first part of this experiment comprised sucrose (%w/w) at 0, 0.05, 0.10, 0.20, 0.40, 0.60, 0.80 and 1.0%, together with 1.86% nicotine, 89.14% VG, and water ranging from 9% for 0% sucrose to 8% for 1.0% sucrose. E-liquids containing 1% glucose or fructose in place of the sucrose were prepared with the same formulation as the 1% sucrose solution. E-liquids used in the second part of the experiment comprised sucrose at concentrations (w/w) of 0, 1, 2.5, 5, 7.5 and 10%. The solutions contained 9% water, 1.86% nicotine and levels of VG ranging from 79.14% for the 10% sucrose solution to 89.14% for the 0% sucrose solution.

E-liquids containing 0–10% sucrose were analysed by Labstat International (Kitchener, Ontario, Canada) for sucrose and carbonyls and dicarbonyls that might potentially contribute to ketone presence in the aerosol. For aerosol analysis a 30W eLeaf iStick e-cigarette was used to generate the aerosol. The puffing parameters were a 80 ml puff volume, 3 s puff duration and 30 s interval. The device was tested at two power settings (10 and 20 W) to observe whether increased power influenced yields. Blocks of 50 puffs were collected for analysis. For both series of experiments, machine puffing, aerosol collection and analytical testing were conducted by Labstat International (Kitchener, Ontario, Canada). Labstat method TMS-00155 (Carbonyls and Dicarbonyls) were used for these studies (Bao 2015) as described in the previous section.

### Pyrolysis Screening Experiments for AC, AP and DA From Flavor Compounds

A series of five- and six-membered ring flavor compounds were investigated in the pyrolysis experiments. Some samples consisted of “neat” material and some were solutions in 3:1 VG:PG (by weight). PG and VG were also examined in the pyrolysis study to provide comparability with the other compounds of this study, and also to provide baseline levels of DA or AP for those experiments in which they were used as carriers. The flavor compounds investigated were furaneol (2,5-dimethyl-4-hydroxy-3(2H)-furanone) tested as both a 5% solution in 3:1 VG:PG and 15% in PG; 5-ethyl-3-hydroxy-4-methyl-2(5H)-furanone tested as both a 5% solution in 3:1 VG:PG and as the neat compound; mesifurane (2,5-dimethyl-4-methoxy-3(2H)-furanone) tested as the neat compound; furaneol acetate (2,5-dimethyl-4-acetoxy-3(2H)-furanone) tested as the neat compound; ethyl maltol (3-hydroxy-2-ethyl-4-pyranone) tested as the neat compound and as a 5% solution in VG; cyclotene (3-methyl-2-cyclopenten-2-ol-1-one) tested both as a 5% solution in VG and neat; 1,8-cineole (1,3,3-trimethyl-2-oxabicyclo[2,2,2]octane) tested as the pure compound; vanillin (4-hydroxy-3-methoxybenzaldehyde) tested neat; ethyl vanillin (4-hydroxy-3-ethoxybenzaldehyde) tested neat; 4-ketoisophorone (2,6,6-trimethylcyclohex-2-ene-1,4-dione) tested neat; β-damascone ((E)-1-(2,6,6-trimethyl-1-cyclohexenyl)but-2-en-1-one) tested neat, and peppermint oil (mix of menthol (7–48%), menthone (20–46%), menthyl acetate (3–10%), menthofuran (1–17%), 1,8-cineole (3–6%), etc.) tested neat.

The samples were pyrolyzed using a filament pyrolyzer Pyroprobe 5000 Model 520 equipped with autosampling capability from CDS Analytical INC. (Oxford, PA 19363, United States). All materials were loaded on a fiberglass bed at a specific amount (around 2 mg material precisely weighed). The pyrolysate was directly transferred to a 6890/5973 GC/MS from Agilent (Wilmington, DE 19808, United States). This system was used in flash mode with pyrolysis performed in the carrier gas (helium). Except for the pyrolysis temperature, other conditions for the pyrolysis were kept the same in all experiments: purge time *t* = 0.0 s, equilibration time, *t* = 0.0 s, pyrolysis time 40 s, post pyrolysis time *t* = 12 s, valve temperature 250°C, transfer line temperature 250°C. The temperatures for each sample were set at specific values such as 250°C, 350°C, 450°C and in a few cases at 550°C. The higher temperatures were not expected to be attained in e-cigarettes and the pyrolysis at these temperatures was performed only for verifying an expected increase in the level of pyrolytic products. The GC/MS parameters for the separation are described in Supplemental Table S3. During data analysis, peak identification used the Wiley275 and NIST14 mass spectral libraries. Retention times and spectra for DA and AP were identified using 2% solutions in acetone.

### Statistical Testing and LOQ/LOD Results

Statistical analysis of the data from all the studies was performed using Minitab version 16 (Minitab Inc, PA, United States) and Minitab version 20. Tests of significance were carried out using one-way analysis of variance at a confidence level of 95%. Comparisons were made with Tukey’s method. For graphical presentation, where results were LOD, values of LOD/2 were used, and where results were LOQ, values were assigned as LOD+(LOQ-LOD)/2. Regression analyses were conducted using the Minitab v20 regression assistant.

## Results

### Transfer of AC, AP and DA From e-Liquids to Aerosols

Results of the experiments to determine the effects of storage time on the concentrations of AC, AP and DA in e-liquids spiked with 1,000 μg/ml of these chemicals, and their corresponding aerosol emissions are shown in Tables 1-3. The e-liquid results (but not the aerosol emission data) have been reported previously (Vas et al., 2019).

**TABLE 1:** Transfer of AC from e-liquid to e-cigarette aerosol.

Time (Days)	Device weight loss (mg/100 puffs)		E-liquid [AC] (µg/ml)		Aerosol AC emissions µg/100 puffs		AC transfer efficiency (%)	E-liquid [DA] (µg/ml)		Aerosol DA emissions µg/100 puffs		DA transfer efficiency (%)
	Mean	SD	Mean	SD	Mean	SD		Mean	SD	Mean	SD	
Control	303	40	<1.87	—	<2.08	—	—	<1.13	—	<1.79	—	—
0	323	61	1,169	46	281	50	83.6	2.36	0.06	<1.79	—	—
3	339	41	1,054	8	286	36	89.9	13.7	0.2	2.88	1.88	69.6
6	374	22	1,034	14	329	26	95.5	17.1	0.3	3.15	0.56	55.3
9	364	39	963	13	295	44	94.5	19.6	0.3	4.70	1.2	74.0
12	371	9	963	10	302	10	94.9	21.2	0.8	11.0	0.5	157.1
15	358	29	1,059	14	270	25	80.0	26.5	0.9	11.1	2.2	131.4
18	504	26	981	20	396	6.93	89.9	29.3	0.7	15.0	0.074	114.1
21	453	12	905	14	347	28	95.1	29.6	0.1	14.1	1.0	118.1
64	460	13	760	6	339	16.1	108.9	46.4	0.4	16.5	2.38	86.8
Mean % Transfer	—	—	—	—	—	—	92.5 ± 8.2	—	—	—	—	101 ± 34.9

Control samples run at days 0 and 36 showed no detectable levels of AC, AP or DA in the control e-liquid or corresponding aerosol sample. AP was not found at quantifiable levels in any e-liquid or aerosol sample, other than aerosol emissions of 5 µg/100 puffs at *T* = 0 days. ± values presented are ±1 standard deviation.

**TABLE 2:** Transfer of diacetyl from e-liquid to e-cigarette aerosol.

Time (Days)	Device weight loss (mg/100 puffs)		E-liquid [AC] (µg/ml)		Aerosol AC emissions µg/100 puffs		E-liquid [DA] (µg/ml)		Aerosol DA emissions µg/100 puffs		DA transfer efficiency (%)
	Mean	SD	Mean	SD	Mean	SD	Mean	SD	Mean	SD	
Control	303	40	<1.87	—	<2.08	—	<1.13	—	<1.79	—	—
0	280	95	<1.87	—	8.56	11.2	1,114	15	245	82	88.2
6	333	62	<1.87	—	18.6	8.3	603	3	115	15	64.3
12	360	30	<0.751	—	2.08	0.00	348	18	68.3	7.2	61.2
18	483	69	<0.751	—	28.1	12.7	366	2	76.3	11.4	48.5
24	556	2	<1.87	—	<2.08	—	240	3	66.1	0.6	55.6
30	552	33	<1.87	—	<1.72	—	190	1	59.4	4.6	63.6
36	524	43	<1.87	—	<1.72	—	164	3	47.6	3.9	62.2
Mean % Transfer	—	—	—	—	—	—	—	—	—	—	63.4 ± 12.3

Control samples run at days 0 and 36 showed no detectable levels of AC, AP or DA in the control e-liquid or corresponding aerosol sample. AP was not found at quantifiable levels in any e-liquid or aerosol sample. ± values presented are ±1 standard deviation.

**TABLE 3:** Transfer of acetyl propionyl from e-liquid to e-cigarette aerosol.

Time (Days)	Device weight loss (mg/100 puffs)		E-liquid [AP] (µg/mL		Aerosol AP emissions µg/100 puffs		AP transfer efficiency (%)	E-liquid [DA] (µg/ml)		Aerosol DA emissions µg/100 puffs		DA transfer efficiency (%)
	Mean	SD	Mean	SD	Mean	SD		Mean	SD	Mean	SD	
Control	303	40	<1.87	—	<2.08	—	—	<1.13	—	<1.79	—	—
0	386	6	522	9	158	24	88.1	4.38	0.09	<1.79	—	—
3	356	19	135	1	27.6	1.7	64.5	3.49	0.09	<1.79	—	—
6	342	39	92.3	2.8	20.8	1.8	74.0	2.94	0.14	<1.79	—	—
9	286	114	64.2	4.6	14.3	4.8	87.5	1.40	0.08	<1.79	—	—
12	369	9	60.5	2.8	12.3	0.4	61.9	1.26	0.05	<1.79	—	—
15	405	170	55.0	0.8	14.0	5.1	70.6	0.869	0.070	<1.79	—	—
18	491	104	70.3	0.7	13.8	1.7	44.9	0.861	0.011	<1.79	—	—
21	558	7	60.5	1.4	13.6	0.5	45.2	0.581	0.070	<1.79	—	—
64	416	149	38.5	0.8	<10.3	—	—	0.977	0.017	<4.23	—	—
Mean % Transfer	—	—	—	—	—	—	67.1 ± 16.6	—	—	—	—	—

Control samples run at days 0 and 36 showed no detectable levels of AC, AP or DA in the control e-liquid or corresponding aerosol sample. AC was not found at quantifiable levels in any e-liquid or aerosol sample. ± values presented are ±1 standard deviation.

Table 1 shows that e-liquid AC concentrations fell significantly (*p* < 0.005) with increasing time, with an [AC]_liquid_ 35% lower after 64 days than the day 0 value. In contrast, aerosol AC emissions from the e-cigarettes did not appear to change significantly over time. However, allowing for variation in device mass loss, i.e. total amount of aerosol generated by the e-cigarettes over the course of the experiment, showed the DML-normalized [AC]_aerosol_ values did decline significantly (*p* = 0.005) by around 16% over the 64 day time course of the experiment. As reported previously (Vas et al., 2019) the AC-containing e-liquid generated DA. Levels of DA in both e-liquid and aerosol increased significantly (*p* < 0.001) over time. Transfer efficiency of AC from e-liquid to aerosol was near-quantitative, at 92.5 ± 8.2%, while the transfer efficiency of DA generated from AC e-liquid was quantitative, albeit highly variable, at 101 ± 34.9%.

The data in Table 2 show that DA levels in e-liquids and aerosol fell significantly over time (*p* < 0.05). [AC]_liquid_ fell by 85% over the 36 day experiment, and [AC]_aerosol_ fell by 80% (90% when normalized to DML). These changes were substantially greater than found with AC. DA transfer efficiency was 63.4 ± 12.3%, with the first time point providing a % transfer efficiency significantly higher than found at the other time points (*p* < 0.05). Interestingly, for half of the time points relatively low-level AC emissions were detected in the aerosol of the DA e-liquid, despite their absence from the e-liquid at any time point. The aerosol AC levels were greater than the e-liquid detection limit, and these levels of AC would have been detected in the e-liquid if present.

Table 3 shows that [AP]_liquid_ fell over the 64-day experiment to approximately 4% of the amount added to the e-liquid. Aerosol AP levels fell in a very similar way, reaching non-quantifiable levels after 64 days (<7% of the day 0 value). Transfer efficiency of AP from e-liquid to aerosol was similar to the value found with DA, at 67.1 ± 16.6%. Small quantities of DA were found in the AP e-liquid but transfer of the DA to the aerosol was too low to quantify.

Regression analysis of aerosol emissions against e-liquid content showed significant correlations (*p* < 0.001) for both AP and DA, with 98.2% of the variation in the AP data and 82.4% of the variation in the measured DA emissions accounted for by the e-liquid contents of these compounds. Multiple regression of the DA emissions against both the DA e-liquid content and the DML raised the *r* ^2^ value to 98.6%, but the corresponding analysis for the AP data did not change the *r* ^2^ value from the 98.2% provided by the simple regression against e-liquids AP concentration. In contrast, the aerosol AC emissions were not significantly correlated with the e-liquid AC levels (*r* ^2^ = 12.2%, *p* = 0.07). However, multiple regression of aerosol AC emissions against both e-liquid [AC] and DML showed significant (*r* ^2^ = 87.2%, *p* < 0.001) correlation.

### The Potential of e-Liquid VG and PG to Generate AC, AP and DA in e-Cigarette Aerosols

Results from experiments examining the potential thermal formation of AC, AP and DA from VG, PG and 1,3 PD are shown in Table 4. The table shows the power setting, resulting coil temperature, per-puff aerosol yields of AC, AP and DA, and e-cigarette total mass loss for the three glycol solutions tested. Supplementary Table S2 shows the LODs and LOQs for the method.

**TABLE 4:** Effect of power setting on aerosol yields of acetoin, acetyl propionyl and diacetyl from e-cigarettes with e-liquids consisting of mixtures of humectants and water.

Formulation	Setting	Temperature	Aerosol yield [ng/puff]			Mass loss
	(W)	(°C)	Acetoin (Mean ± SD)	Acetyl propionyl (Mean ± SD)	Diacetyl (Mean ± SD)	(mg/collection)
VG 75% Water 25%	10	248	<LOD^0^	<LOD^0^	<LOD^0^	198
	12	259	<LOD^0^	<LOD^0^	<LOD^0^	225
	14	254	<LOD^0^	<LOD^0^	<LOD^0^	300
	16	254	<LOD^0^	<LOD^0^	<LOD^0^	403
	18	262	<LOD^0^	<LOD^1^	<LOD^1^	446
	20	257	<LOD^0^	<LOD^0^	<LOD^0^	493
	25	247	<LOD^0^	<LOD^0^	<LOD^0^	661
	26.5	265	<LOD^0^	<LOD^0^	53.6 ± 97.3	727
	27.5	278	<LOD^0^	<LOD^0^	<LOQ^1^	783
	28.5	293	<LOD^0^	<LOD^0^	26.3 ± 33.3	801
	30	302	<LOD^1^	<LOD^1^	31.1 ± 42.1	842
	32	359	<LOD^0^	45.2 ± 20.3	204.7 ± 72.7	1,057
	35	394	83.6 ± 122.4	87.8 ± 55.6	369.4 ± 218.6	883
1,3 PD 91% Water 9%	10	204	<LOD^0^	<LOD^0^	<LOD^0^	309
	12	215	<LOD^0^	<LOD^0^	<LOD^0^	368
	14	219	<LOD^0^	<LOD^0^	<LOD^0^	436
	16	220	<LOD^0^	<LOD^0^	<LOD^1^	565
	18	225	<LOD^0^	<LOD^0^	<LOD^0^	622
	20	238	<LOD^0^	<LOD^0^	<LOD^0^	624
	25	335	<LOD^0^	<LOD^0^	<LOD^0^	764
	26.5	382	<LOD^0^	<LOD^0^	<LOD^1^	1,048
	27.5	398	<LOD^0^	<LOD^0^	<LOD^1^	1,083
	28.5	387	<LOD^0^	<LOD^0^	<LOD^1^	1,113
	30	363	<LOD^0^	<LOD^0^	12.4 ± 12.1	976
	32	404	<LOD^0^	29.4 ± 47.3	<LOQ^3^	1,331
	35	441	<LOD^0^	45.7 ± 77.0	14.1 ± 13.9	1,177
PG 91% Water 9%	10	185	<LOD^0^	<LOD^0^	<LOD^0^	327
	12	182	<LOD^0^	<LOD^0^	<LOD^0^	368
	14	181	<LOD^0^	<LOD^0^	<LOD^0^	515
	16	180	<LOD^0^	<LOD^0^	<LOQ^1^	594
	18	180	<LOD^0^	<LOQ^2^	17.1 ± 18.8	650
	20	179	<LOD^0^	<LOD^0^	<LOD^0^	564
	25	233	<LOD^0^	<LOD^0^	<LOD^0^	725
	26.5	244	<LOD^0^	<LOD^0^	<LOD^2^	816
	27.5	234	<LOD^0^	<LOD^0^	12.1 ± 12.7	828
	28.5	ND	<LOD^0^	<LOD^0^	22.2 ± 33.1	969
	30	325	<LOD^0^	<LOQ^1^	55.4 ± 46.1	810
	32	417	<LOD^0^	<LOQ^2^	87.9 ± 94.0	825
	35	452	<LOD^0^	<LOQ^2^	125.0 ± 61.7	1,254

ND, not determined. ^0, 1, 2, 3^ Numbers of replicates (out of 5) with values >LOD. ± values presented are ±1 standard deviation.

The device mass loss of the e-cigarette (per 25 puffs), i.e. the weight of aerosolized e-liquid, increased as the power to the coil increased. This is illustrated in Supplementary Figure S1. The relationship between power and mass loss appears linear for all three of the e-liquids studied, with correlation coefficients (*r* ^2^) of 0.862, 0.928 and 0.956 and for PG, 1,3-PD and VG containing e-liquids, respectively. The mass loss/unit power is highest for 1,3 PD, lowest for VG and intermediate for PG.

Coil temperatures, as measured by thermocouple, generally increased with power for the three e-liquids studied but the relationship was not linear and each of the three e-liquids gave a different pattern, as shown in Supplementary Figure S2. As power was increased from 10 to 20 W, the coil temperatures remained fairly constant for both VG (at about 255 ± 7°C) and PG (at about 182 ± 3°C). For 1,3 PD, coil temperatures increased gradually from about 200 to 240°C as power was increased. At 25 W there were sharp increases in coil temperature for both PG (up to 233°C) and 1,3-PD (up to 335°C). As power was increased to a maximum of 35 W, coil temperatures increased up to 452°C for PG and 441°C for 1,3 PD. However, there were shoulders in the power/temperature curves at 26.5 W/244°C for PG and at 27.5 W/398°C for 1,3 PD. For VG the coil temperature remained at about 250°C for power inputs of up to about 25 W and thereafter rose monotonically up to a maximum of 394°C at 35 W.

**FIGURE 2:**
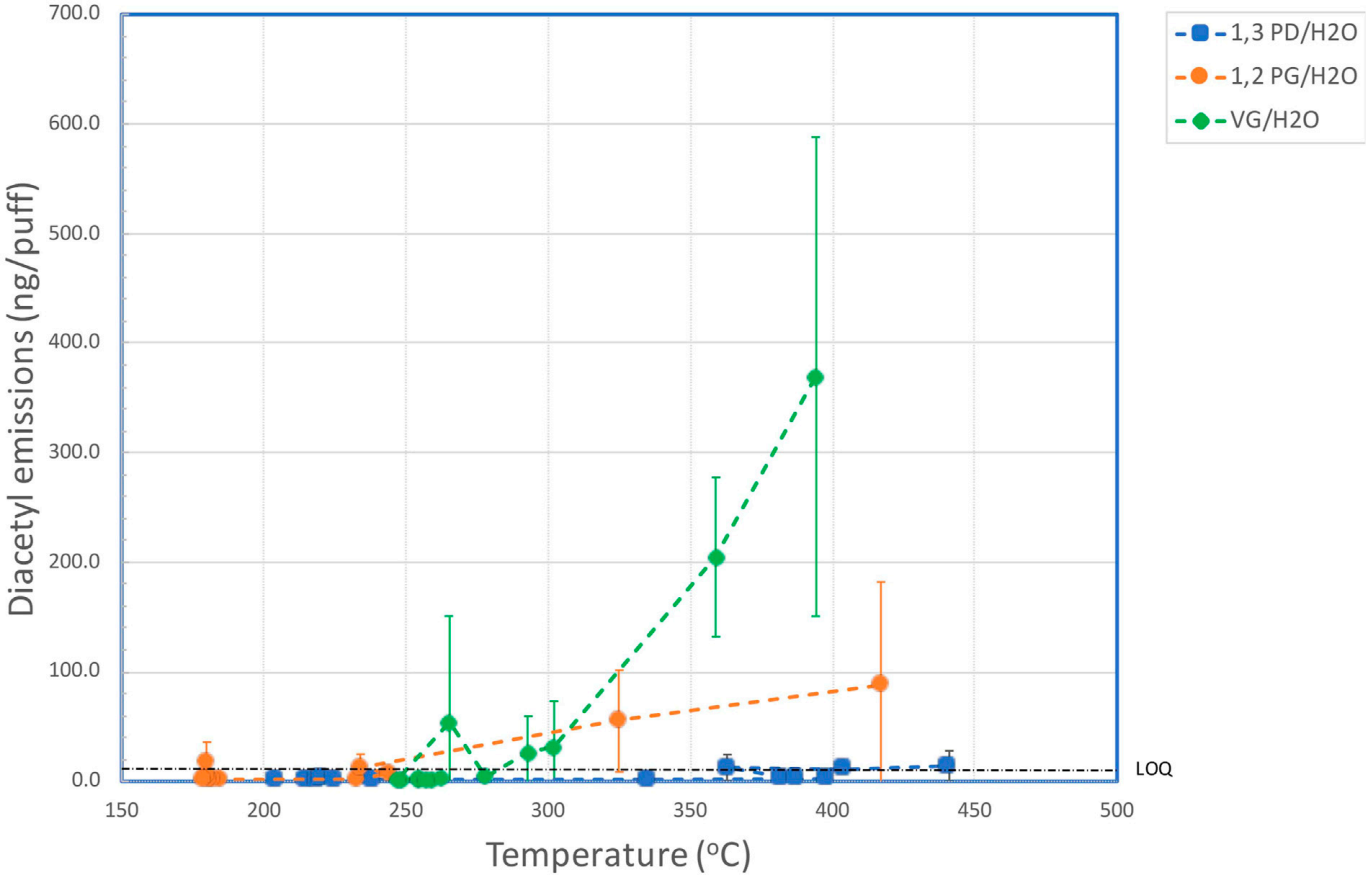
Aerosol yields of diacetyl versus coil temperature for aqueous solutions of VG, PG and 1,3-PD.

Table 4 shows the effect of coil temperature on AC emissions (Supplementary Figure S3), AP emissions (Supplementary Figure S4), and DA emissions (Figure 2).

Levels of aerosol AC produced by the PG and 1,3 PD e-liquids were all below the LOD (0.0134 µg/puff) for coil temperatures up to around 450°C. The VG-containing e-liquid produced only one value for AC above the LOQ (0.045 µg/puff) and that was 0.084 µg/puff at the maximum coil temperature of 394°C. However, this value was not significantly different (*p* > 0.05) to the results obtained at lower temperatures, with only two of the five replicates showing values of AC > LOQ.

AP was detected in the aerosol from PG at various temperatures but levels never exceeded the LOQ (0.0235 µg/puff) even at the highest coil temperature (452°C). For 1,3-PD, levels of AP in the aerosol remained below the LOD (0.007 µg/puff) until coil temperatures exceeded 400°C. Quantifiable levels of AP were observed at the two highest coil temperatures achieved: 0.029 µg/puff at 404°C and 0.046 µg/puff at 441°C. These yields were not significantly greater (at 95%) than those generated at lower temperatures. For VG, levels of AP were below LOD up to 302°C, but quantifiable levels of AP were measured at the two higher coil temperatures: 359°C (0.045 µg/puff) and 394°C (0.088 µg/puff), with the latter yield significantly greater (at 95%) than that at 302°C.

The yields of DA as a function of coil temperature for the three e-liquids are shown in Figure 2. For 1,3 PD, levels of DA in the aerosol were <LOD for coil temperatures up to 335°C, but quantifiable levels of DA were observed for the aerosol generated at 363°C and 441°C. These emissions were not significantly different (at 95%) to those at lower temperatures. With PG quantifiable levels of DA were observed with one measurement at 180°C and for coil temperatures above 234°C. Levels of DA increased with coil temperature with the highest level of DA (0.125 µg/puff) observed at the maximum coil temperature of 452°C. With VG, levels of DA increased rapidly at coil temperatures above 293°C. The level of DA in the aerosol at the highest coil temperature (394°C) was 0.369 µg/puff, which was significantly greater than the yield at 302°C.

### Potential of Sugars in e-Liquids to Generate Aerosol AC, AP and DA

E-liquid analysis showed that apart from sucrose there were detectable levels of formaldehyde (mean 1.56 μg/g), glycolaldehyde (mean 1.153 μg/g), AC (mean 0.565 μg/g), glyoxal (mean 0.991 μg/g) and methylglyoxal (mean 2.61 μg/g). These levels were all greater than the laboratory reagent blank (LRB) and the concentrations were similar for all the formulations. Acetone was also detected in all the formulations (mean 0.657 μg/g) but the levels in the LRB were not significantly different to the test liquids which indicates acetone was introduced via the analytical procedure. There were no detectable levels of the other analytes included in the assay: acetaldehyde, propionaldehyde, acrolein, isobutyraldehyde, methyl ethyl ketone, 3-buten-2-one, n-butyraldehyde, crotonaldehyde, acetol, DA, AP, 2,3-hexanedione or 2,3-heptanedione.

**FIGURE 3:**
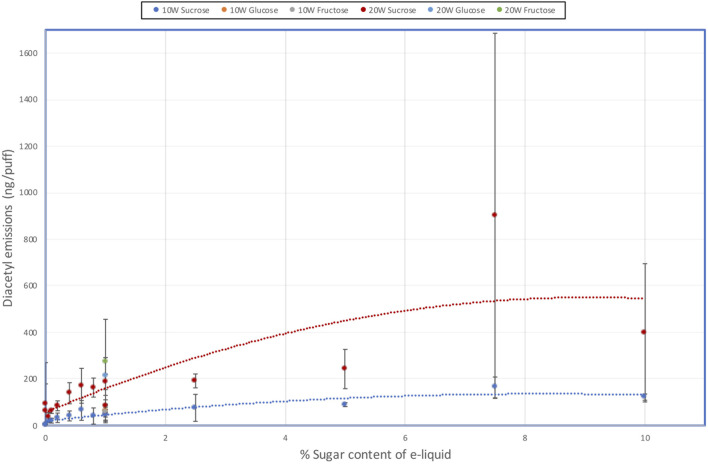
Effect of e-liquid sugar levels on DA emissions from an e-cigarette at 10 W and 20 W power.

The results showing the concentrations of DA and AP in the aerosols of the e-liquids are shown in Table 5 and DA emissions illustrated in Figure 3. AC concentrations were all below the LOD and are therefore not shown.

**TABLE 5:** Device mass losses and aerosol yields of AC^a^, AP and DA per puff at power settings of 10 and 20 W, from 0 to 10% sucrose solutions.

Formulation (%)						DML @10 W mg/puff	DA emissions @10 W (ng/puff)		AP emissions @10 W (ng/puff)		DML @20 W mg/puff	DA emissions @20 W (ng/puff)		AP emissions @20 W (ng/puff)	
Sucrose	Fructose	Glucose	Glycerol	Water	Nicotine	Average	Average	St Dev	Average	St Dev	Average	Average	St Dev	Average	St Dev
0.00^b^			89.14	9.00	1.86	6.76	NQ	NQ	BDL	BDL	24.40	64.00	114.80	15.16	29.98
0.05^b^			89.14	8.95	1.86	8.18	15.04	2.22	BDL	BDL	25.40	37.20	18.40	NQ	NQ
0.10^b^			89.14	8.90	1.86	7.66	20.60	10.02	BDL	BDL	23.70	60.60	9.00	NQ	NQ
0.20^b^			89.14	8.80	1.86	10.80	33.00	20.40	NQ	NQ	23.70	84.40	21.20	NQ	NQ
0.40^b^			89.14	8.60	1.86	10.50	41.00	21.40	NQ	NQ	24.70	138.60	45.20	13.52	8.58
0.60^b^			89.14	8.40	1.86	9.66	64.60	42.60	NQ	NQ	23.90	170.60	75.00	22.20	10.40
0.80^b^			89.14	8.20	1.86	7.05	40.40	34.80	NQ	NQ	24.70	162.80	41.00	19.28	8.58
1.00^b^			89.14	8.00	1.86	9.93	45.20	33.60	BDL	BDL	24.10	187.60	33.40	21.20	8.00
	1.00^b^		89.14	8.00	1.86	8.18	57.40	34.40	NQ	NQ	25.20	272.00	184.00	32.00	29.80
		1.00^b^	89.14	8.00	1.86	7.70	61.20	25.00	NQ	NQ	24.90	210.00	82.00	14.84	11.30
0.00^c^			89.14	9.00	1.86	7.69	NQ	NQ	BDL	BDL	26.24	92.64	177.22	NQ	NQ
1.00^c^			89.14	8.00	1.86	6.20	43.53	25.77	NQ	NQ	25.10	83.20	26.96	BDL	BDL
2.50^c^			86.64	9.00	1.86	5.58	75.68	57.96	BDL	BDL	24.82	191.52	29.86	17.50	17.43
5.00^c^			84.14	9.00	1.86	6.74	89.44	8.28	11.71	8.80	23.58	242.56	84.45	19.29	24.18
7.50^c^			81.64	9.00	1.86	6.17	163.36	44.78	25.69	33.02	28.14	900.80	784.38	16.76	33.56
10.0^c^			79.14	9.00	1.86	5.47	121.76	13.31	17.53	21.61	25.72	398.09	296.98	33.31	20.79

DML, Device Mass Loss; NQ, not quantifiable; BDL, below detection limit.

a AC emissions were BDL for all samples, and therefore not shown.

b Data for the 0–1% sugar experiment.

c Data for the 0–10% sugar experiment.

Aerosol DA emissions from the sucrose-free e-liquids were < LOQ (0.0058 µg/puff) at 10 W, but quantifiable (0.064–0.093 µg/puff) at 20 W; AP emissions were <LOD or <LOQ. With the e-liquids containing sugars there were increases in DA emissions as both the sucrose concentration and heating coil power increased, although there was some scatter in the data. At 10 W, DA yields increased up to 0.16 µg/puff at 7.5% sucrose and then tapered off to 0.12 µg/puff at 10% sucrose. At 20 W DA yields reached 0.90 µg/puff at 7.5% sucrose and then dropped to 0.40 µg/puff at 10% sucrose. DA emissions from the 1% fructose and 1% glucose e-liquids at both power levels were quantifiable and not significantly different to those from the corresponding 1% sucrose containing e-liquid.

AP yields were found to be at significantly lower levels than the DA emissions, but AP emissions also increased as sucrose concentration and power increased. At 10 W, AP emissions were only quantifiable at and above 5% sucrose. At 20 W coil power, quantifiable yields of AP were observed for sucrose concentrations of 0.4% and above, other than one of the 1% sucrose solution measurements. AP emissions from the 1% fructose and 1% glucose e-liquids at 20 W power levels were quantifiable and not significantly different to those from the corresponding 1% sucrose containing e-liquid.

When the e-cigarette was disassembled after analysis, considerable char formation was observed on the coil and wick. The observable char level increased with increasing sugar level but was variable from device to device. This may have contributed to the scatter in the results for both DA and AP analyses.

### Pyrolysis Screening Study

**FIGURE 4:**
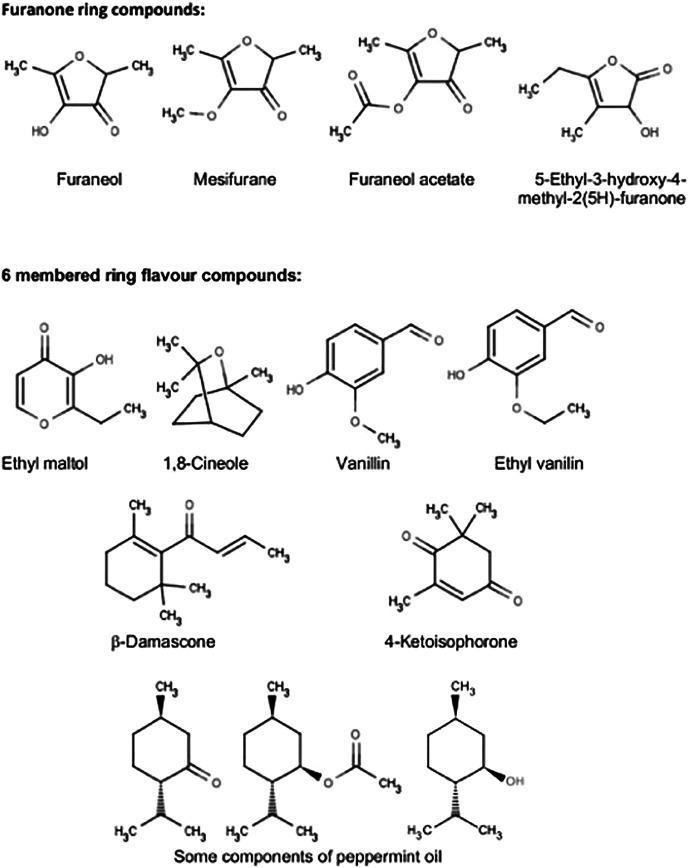
Flavour compounds examined in the pyrolysis screening study.

The structures of the compounds investigated in the pyrolysis experiments are presented in Figure 4, and the results of these experiments are shown in Table 6 for both DA and AP. Table 6 is constructed so as to indicate the presence or absence of the compounds in a specific pyrolysis experiment at a specific temperature. When the presence of DA or AP was detected in the pyrolyzates, it was at an extremely low level, and the diketones were not, by far, the major decomposition products of the pyrolyzed compound. The levels of DA and AP were generally 10^6^–10^9^ times less than the parent pyrolyzed compound. The results from Table 6 describe specific behavior upon heating as follows:

**TABLE 6:** Presence or absence of acetyl propionyl or diacetyl in pyrolysis screening experiments on flavor compounds.

Compound	250°C		350°C		450°C		550°C	
	DA	AP	DA	AP	DA	AP	DA	AP
VG	N	N	N	N	Y	T	Y	Y
PG	N	N	N	N	T	N	T	T
Furaneol in PG + VG	Y	Y	Y	Y	Y	Y	Y	Y
Furaneol 15% in PG	Y	Y	Y	Y	Y	Y	Y	Y
EHM-furanone in PG + VG	N	T	N	Y	Y	Y	—	—
EHM-furanone (neat)	T	Y	Y	Y	—	—	—	—
Mesifurane (neat)	Y	Y	Y	Y	—	—	—	—
Furaneol acetate (neat)	Y	Y	Y	Y	—	—	—	—
Ethyl maltol (neat)	—	—	N	N	N	N	—	—
Ethyl maltol + VG	—	—	N	N	T	T	—	—
Cyclotene in PG + VG	N	N	N	N	T	T	—	—
Cyclotene (neat)	—	—	N	N	N	N	—	—
1,8-Cineole (neat)	—	—	N	N	N	N	—	—
Vanillin (neat)	—	—	N	N	N	N	—	—
Ethyl vanillin (neat)	—	—	N	N	N	N	—	—
Vanillin PG acetal (neat)	—	—	N	N	N	N	—	—
Ethyl vanillin PG acetal (neat)	—	—	N	N	N	N	—	—
4-Ketoisophorone (neat)	—	—	N	N	N	N	Y	Y
ß-Damascone (neat)	—	—	N	N	—	—	—	—
Peppermint oil (neat)	—	—	N	N	N	N	T	T

Y, present; N, not found; T, trace present in chromatogram

#### Aerosol Formers

VG did not generate either DA or AP in the pyrolysis experiments when heated at temperatures up to 350°C. However, when heated at higher temperatures traces of the two compounds were observed, with greater levels of formation at higher temperatures, consistent with Figure 2. PG was more stable than VG to heating, however, traces of DA and AP were also found at temperatures starting at 450°C.

#### Furanose Ring Compounds

Furaneol was found to form DA and AP when pyrolyzed at temperatures as low as 250°C. Mesifurane was even more unstable to heating than furaneol, and both DA and AP formation was observed at 250°C. Furaneol acetate was also more unstable to heating than furaneol and formed DA and AP starting at 250°C. 5-ethyl-3-hydroxy-4-methyl-2(5H)-furanone (EHM-furanone in Table 6) was more stable compared to furaneol and formed DA only when heated to about 450 ^o^C. However, the formation of AP from 5-ethyl-3-hydroxy-4-methyl-2(5H)-furanone started as low as 250°C (in traces) and the AP yield increased at higher temperatures.

#### Six-Membered Ring Flavor Compounds

Ethyl maltol, cyclotene, 1,8-cineole, vanillin, ethyl vanillin, vanillin PG acetal, ethyl vanillin, and PG acetal did not form DA or AP (when pyrolyzed at temperatures up to 450 °C), and ß-damascone did not form DA or AP (at least up to 350°C). Similarly, 4-ketoisophorone formed DA and AP at 550°C but not lower temperatures. The sample of 4-ketoisophorone evaluated in this study also showed a trace of 2,4-pentanedione in the GC trace. Traces of DA and AP were detected in the pyrolyzate of peppermint oil at 550°C but not at lower temperatures.

## Discussion - Sources of AC, AP and DA in E-cigarette Aerosols

### Direct Transfer From e-Liquids

Transfer of these species from e-liquids in which they are present to e-cigarette aerosols were found to be less than 100%, other than the case of DA formed by AC in e-liquids. Transfer of the hydroxyketone AC was greater (92.5%) than found with the di-ketones DA (63.4%) and AP (67.1%). This is a similar finding to the relative stabilities of the three species found when they were stored in nicotine-containing e-liquids (Vas et al., 2019). The only other study to report transfer levels of DA and AP from e-liquids to e-cigarette aerosols was that of Farsalinos et al. (2015), who conducted limited transfer experiments (three data points) without the extended e-liquid storage times of the present study. Farsalinos et al. (2015) reported near-quantitative transfer of AP and DA to the aerosol. Using the regression equations reported by the authors and the 1,000 μg/ml initial e-liquid concentrations of the present study, suggests transfer efficiencies of 83% for AP and 86% for DA. These estimates are higher than the values reported in the present study, but the test e-liquid used by Farsalinos et al. (2015) did not contain nicotine. The reactivity of AP and DA in e-liquids has been shown to be strongly enhanced by basic materials such as nicotine (Vas et al., 2019), and it is highly plausible that the presence of nicotine in the current study e-liquids would have led to the lower stabilities of AP and DA found here.

### Thermal Generation From Major e-Liquid Constituents

In the present study, increasing e-cigarette power from 10 to 20 W was found to produce a fairly constant coil temperature of about 255°C for VG/water (VG B.Pt. 290°C). The coil temperature was also relatively constant at about 182°C with PG/water (PG B.Pt. 188°C). For 1,3 PD/water (1,3 PD B.Pt. 213°C) coil temperatures increased from about 200 to 240°C as power was increased. The relatively steady temperatures at these lower power settings, at around or just below the boiling point of the pure polyol, indicates that sufficient liquid is reaching the coil for stable aerosolisation. The sharp increases in temperature observed at higher power settings probably indicate that the e-liquids can no longer stabilize the coil temperature and overheating is occurring within the atomizer (Geiss et al., 2016).

The levels of AC in the aerosol did not increase significantly for any of the e-liquids up to the maximum coil temperatures achieved, although a single quantifiable value was recorded with VG/water at 394°C. Thermal production of acetoin from these three aerosol formers does not therefore appear to be a viable process in e-cigarettes.

In contrast, emissions of AP were quantifiable from the VG-containing and 1,3-PD-containing e-liquids at the highest coil temperatures, although only the emissions from the VG/water e-liquid showed significant increases over the lower temperature values. VG also had a greater potential to generate AP (and DA) compared to PG and 1,3 PD even though there was a lower concentration of VG (75%) in the e-liquid tested, compared with the concentrations of 91% for both PG and 1,3 PD. DA was generated at coil temperatures over 293°C for VG/water and over 234°C for PG/water. However, DA emissions from 1,3-PD/water were not significantly different from baseline values at up to 441°C.

In the experiments with sugar containing e-liquids, quantifiable yields of DA and AP were observed at 20 W (but not 10 W) from the control e-liquids with no sugar content. One possibility for this observation is that one or more of the contaminants in the solution may have contributed to the DA in the aerosol. DA and AP were not detected in the e-liquid, thereby removing the possibility of direct transfer as the source of these compounds. Alternatively, AC might be oxidised to DA during aerosol formation, however the concentration of AC in the e-liquid (0.565 μg/g) would contribute less than 0.0015 µg/puff even with all the AC oxidised to DA and with 100% transfer. The other contaminants in the e-liquid have shorter carbon chains than DA and AP and would require an associative reaction to form diketones. Martinuzzi et al., (2014) proposed that methylglyoxal was an intermediate in the formation of DA during gas-phase catalytic dehydration of VG, but the e-liquid methylglyoxal concentration in the present study was too low to account for the DA yield in the aerosol. It is therefore more likely that thermal decomposition of VG itself is the source of DA and AP in the sucrose-free liquids.

Three studies have examined the thermal generation of DA (and one examined AP) from VG or PG (Behar et al., 2016; Sleiman et al., 2016; Melvin et al., 2020) in e-cigarettes. Sleiman et al. collected between one and five puffs, sampled both “early” i.e. between the 1st and 5th puffs, or “late” i.e. between the 30th and 40th puff in their experiment. They identified DA in aerosols generated from neat VG and PG as well as from commercial, flavored e-liquids. VG generated 45 and 179 ng/puff respectively from the “early” and “late” puffs. PG generated 113 and 586 ng/puff. Behar et al., (2016) also identified the presence of DA in the aerosol from e-cigarettes containing DA-free e-liquids. Melvin et al., (2020) conducted model studies using a microwave heater to heat an e-liquid at 180°C for 1–15 min, as well as using the same system to examine temperatures over the range 80–220°C with a 3 min heating time. They found that both VG and PG could generate DA under these conditions, via a thermal degradation mechanism involving hydroxyacetone. The production of DA was accelerated by the presence of nicotine. The authors also compared DA and AP e-liquid contents and aerosol emissions from eight cigalike e-cigarettes, and found increased levels of DA in the aerosol samples over and above the e-liquid levels, but little evidence of increased levels of AP. The authors suggested that thermal generation of DA was occurring, and it followed a different or faster mechanistic pathway to that required to thermally generate AP. Our study results support the findings of Melvin et al., (2020), but also provides more realistic temperature and time conditions to establish the thermal conditions required in an e-cigarette to generate these ketones from the major aerosol formers.

Other authors have noted the formation of DA from the aerosol carriers. For example, studies of gas-phase glycerol dehydration using acid-catalysts have shown that DA can be formed at temperatures of about 300°C. Lauriol-Garbay et al. (2011) vaporised a 20% aqueous glycerol solution with an inert gas flow and passed it over Zr/Nb mixed oxide catalysts at 280–300°C. The major product was AC, but increased concentrations of DA were observed as the temperature increased. Selectivity for production of DA increased with temperature from 0% at 280°C, to 0.3% at 290°C and 0.8% at 300°C. Similarly, Martinuzzi et al. (2014) studied glycerol dehydration at 270–308°C over a solid acid catalyst, with up to 6% oxygen in the gas stream. DA was found as a reaction product with a selectivity ranging from 0.015 to 0.061%. By passing a number of potential intermediates and fragmentation products through the catalytic system, they found that methylglyoxal, an intermediate in the thermal breakdown of glycerol, was a major precursor for DA with a product selectivity of 5%. However, the precise pathway from methylglyoxal to DA was not elaborated.

Together these data confirm that thermal generation of DA and AP from the most common e-cigarette aerosol carriers can occur, providing threshold temperatures are reached. Given the similar temperature profiles noted for formation of DA in the present study and for the catalytic studies described above, we think that the possibility of DA formation from glycerol through a surface reaction on the coil, possibly via methylglyoxal or hydroxyacetone is a hypothesis that is worthwhile investigating in future studies.

### Thermal Generation From Minor e-Liquid Constituents (Flavor Compounds and Sugars)

The transfer experiments of the present study showed the presence of AC in the aerosol from an e-liquid containing DA, despite AC not being detected in the e-liquid. Aerosol AC levels were sufficiently high that they would have been detectable in the e-liquid if they had been present. Data reported by Vas et al., (2019) did not find any conversion of DA to AC at room temperature storage conditions. The simplest explanation for the observation from the present study is to hypothesise thermal reduction of DA to AC at the temperatures found in the e-cigarette atomizer. Further work is needed to investigate this potential mechanism more fully.

Our pyrolysis experiments showed results with PG and VG that were consistent with the findings from our e-cigarette thermal generation studies, despite the differing temperature and time conditions operating in the two studies. A clear finding from the pyrolysis experiments was the relative ease of formation of DA and AP from compounds containing a furanone ring structure, compared to various flavor compounds based on 6-membered ring structures. The importance of ring size identified in this experiment is highlighted by the comparison of furaneol and ethyl maltol, both of which have heterocyclic ring structures with ketone side groups. With furaneol, DA and AP were identified at a pyrolysis temperature of 250°C, whereas pure ethyl maltol did not show evidence for DA and AP formation even at a pyrolysis temperature of 450°C. Five-membered ring compounds are generally regarded as being under greater internal strain than six-membered rings, and it is likely that ring opening can occur at lower temperatures with the furanone ring compounds than with the six-membered ring compounds. The possibility of DA and AP generation from other flavor compounds has received little attention to date, although Behar et al., 2016 found DA was formed as a secondary reaction product of aerosolized e-liquids containing cinnamaldehyde, benzyl alcohol and triacetin. However, the authors did not distinguish between possible formation from flavor compounds or aerosol formers.

Our data also demonstrated that DA and to a lesser extent AP were generated from e-liquids containing sucrose, glucose and fructose. Thermal decomposition of sucrose is well characterised (Monte and Maga 1981) and can take place at temperatures as low as 150–200°C via fragmentation to glucose and fructose with the loss of water. As the temperature increases, caramelisation occurs with the monosaccharides either decomposing to a large variety of smaller molecules including the diketones, DA and AP (Monte and Maga 1981), or oligomerizing to larger molecules with further loss of water. Continued loss of hydrogen and oxygen (as water) eventually leads to the formation of char. There is some debate as to whether the diketones are primarily formed from the backbones of the monosaccharides or from recombination of smaller fragments. A recent study of diketone formation from coffee beans infused with ^13^C-labeled sucrose and roasted at 200°C showed that diacetyl was mostly formed from recombined sucrose C_2_ fragments while AP was formed from the sucrose skeleton (Poisson et al., 2018). Interestingly, glucose is a six-membered ring molecule, and fructose exists in solution as a mixture of the five-membered ring compound ß-D-fructofuranose, and the six membered ring compound ß-D-fructopyranose. Sucrose, fructose and glucose provided similar DA and AP emissions, in contrast to the findings from our pyrolysis experiments of significantly easier production of DA and AP from five-membered ring compounds. The most likely explanation of this is that the flavor compounds examined in our pyrolysis experiments are volatile and can evaporate away from hot metal surfaces, whereas the involatile sugars are unable to leave the heated coil area and thermally decompose. Exposure of sugars to the temperatures of e-cigarette coils at certain power settings appear sufficient to generate diketones. The similarity of diketone emissions from sucrose, fructose and glucose e-liquids supports the mechanism proposed by Monte and Maga (1981) of sucrose thermal degradation proceeding via fructose and glucose production prior to DA/AP generation.

Assessing the likely contribution of sugars to DA and AP emissions from e-cigarettes requires an understanding of the sugar levels found in e-liquids. Kubica et al., (2014) determined sucrose levels in 37 e-liquid samples from seven manufacturers. With a detection limit of 0.73 μg/g, sucrose was found in all the samples with concentrations ranging from 0.76 to 72.93 μg/g. Most (78%) of the samples had less than 20 μg/g of sucrose. The liquids were also analysed for the disaccharides, maltose and lactose, and the monosaccharides, glucose and fructose, but none of the samples contained sugars other than sucrose. Fagan et al., (2017) analysed 66 e-liquids for sugars and aldehydes. With LOQs of 6 μg/ml for glucose and fructose, and 12 μg/ml for sucrose, glucose was quantified in 22% of samples (range: 6.4–88.9 μg/ml, median: <6 μg/ml), fructose in 53% of samples (range: 8.8–331.2 μg/ml, median: 9.7 μg/ml) and sucrose in 53% of samples (range: 9.3–620 μg/ml, median: 18.9 μg/ml).

These two reports show that commercial e-liquids have considerably lower sugar levels than were used in our present study. The lowest level used in our study was 0.05% sucrose, which is equivalent to 600 μg/ml. The highest sugar level found in the Fagan et al. study was 620 μg/ml, which is similar to the lowest level in our study, but the highest level reported by Kubica et al. was 72.9 μg/g sucrose, which is significantly below the lowest level used in our study (0.05%). At the lowest sugar level of our study, DA emissions were 15–37 ng/puff and AP emission were <LOD/<LOQ. Therefore, it appears that while sugar levels reported to be in commercial e-liquids may generate very low levels of DA, AP is unlikely to be generated at measurable levels.

Supplementary Table S4 collates published aerosol emission measurements of AC, AP and DA from commercial e-cigarettes. When available as a per-puff value, the published data is very consistent with the levels found in the experiments of the current study. This suggests that our study findings on the sources of these ketones in e-cigarette aerosols are very relevant to the levels of diketones found with commercial e-cigarettes, and may well point to reasons for the presence of AC, AP and DA in these published studies.

A limitation of the present study is that it was not possible to comprehensively characterize in a single exercise the ketone production potential of all ingredients added to e-cigarettes. While our study identifies some key sources and conditions that lead to ketone production, other potential sources such as other flavour compounds, ethanol and surface reactions at metal coils should also be examined in future studies for their relevance to ketone production during vaping.

## Conclusion

Our results show that AC added to e-liquids is transferred efficiently (>90%) to the aerosol, while transfer efficiencies of AP and DA from e-liquids are lower (ca. 65%), indicating some losses during the thermal processes leading to aerosolisation.

Although thermal degradation of VG can potentially contribute to DA in the aerosol, DA was not detected in aerosols generated at less than 16 W in the e-cigarette used. Significant quantities are only produced at coil temperatures which are much higher than normally achieved during vaping. Quantifiable levels of DA from PG were only found in the aerosol at even higher coil temperatures, while 1,3 PD produced very little DA under any of the conditions studied.

Sucrose, glucose and fructose were also found to generate DA in the aerosols. Quantifiable amounts of DA were found in the aerosol generated at 10W from e-liquids containing sucrose at levels of 0.05%, the lowest concentration studied. DA emissions generally increased with the concentration of the sugar in the e-liquid and with the power supplied to the coil. Our experiments indicated that glucose and fructose have a similar potential to sucrose in generating DA when heated. These observations are important since in addition to direct sugar addition to e-liquids, they may be present in e-liquids if manufacturers incorporate natural fruit and plant extracts. Pyrolysis experiments demonstrated that compounds containing five-membered furanose rings can easily generate AP and DA on heating. Our findings should be considered by manufacturers selecting flavor compounds for use in e-liquids, in order to minimize diketone exposure amongst e-cigarette users.

## Data Availability

The original contributions presented in the study are included in the article/Supplementary Material, further inquiries can be directed to the corresponding author.

## Abbreviations

DA, 2,3 butanedione, diacetyl; AP, 2,3 pentanedione, acetyl propionyl; AC, acetoin; VG, Vegetable glycerol, propane-1,2,3-triol; PG, 1,2-propylene glycol; 1,3 PD, 1,3-propylene diol; NIOSH, National Institute for Occupational Safety and Health; LOD, Limit of Detection; LOQ, Limit of Quantitation; TWA, Time weighted average; STEL, Short term exposure limit.
