# Supplementary material for: Diacetyl and Other Ketones in e-Cigarette Aerosols: Some Important Sources and Contributing Factors
Source: Front Chem. 2021 Sep 23;9:742538. doi: 10.3389/fchem.2021.742538 (PMC8495241; doi:10.3389/fchem.2021.742538)

Supplementary Information:

Supplementary Table 1: Reports of acetoin, acetyl propionyl or diacetyl in e-liquids

| **Reference** | **Experimental** | **Origin of samples** | **DA** | **AP** | **Acetoin** |  | **Comments** |
| --- | --- | --- | --- | --- | --- | --- | --- |
| Farsalinos et al 2015 | 159 e-liquids (refills & concentrates) | Europe and USA | DA in 110 (69%) of samples. Median 29 µg/mL, IQR 10-172 µg/mL. | AP in 53 (33%) of samples. Median values: 44 µg/mL, IQR 7-172 µg/mL. | Not measured |  | Transfer to aerosol quantitative |
| Barhdadi et al 2017 | 12 commercial e-liquids. | Belgium | DA detected in 2/12 and quantified in 2/12 (98.8 & 6.0 µg/g) | AP quantified (10.9 & 8.4 µg/g) in the 2 samples with DA | Not measured |  | Samples obtained 2013-15 |
| Moldoveanu et al. 2017 | 37 commercial e-liquids analysed. | USA | 37 e-liquids had DA in range 0.065-452.6 µg/g. | 37 e-liquids had AP in range 0.026-156.3 µg/g | Not measured |  |  |
| Le Bouf et al 2018 | 146 commercial e-liquids | USA | 67/146 e-liquids had DA in the estimated range 0-106 µg/g | 28/146 e-liquids had AP present | Not measured |  |  |
| Cox et al 2019 | 33 DIY e-liquids | Europe and USA | Not reported | Not reported | Present in 8/33 samples |  |  |
| Czoli et al. 2019 | 166 commercial e-liquids | Ontario, Canada | DA was detected in 2/166 e-liquids | Not reported | Not reported |  | Samples obtained in 2015 |

Supplementary Table 2: LODs and LOQs for the determination of acetoin, acetyl propionyl or diacetyl emissions in the aerosols from the humectant/temperature studies.

|  | LOD | LOQ |
| --- | --- | --- |
|  | ng/puff | |
| Acetoin | 13.4 | 45.0 |
| Acetyl propionyl | 7.0 | 23.5 |
| Diacetyl | 3.5 | 11.6 |

Supplementary Table 3. Parameters for the GC/MS on-line analysis of pyrolysates*.*

| **Parameter** | **Description** | **Parameter** | **Description** |
| --- | --- | --- | --- |
| GC column | DB-1701 | Flow mode | Constant flow |
| Column dimensions | 60 m long, 0.25 mm id. | Flow rate | 1.1 mL/min |
| Film thickness | 1.0 μm | Nominal initial pressure | 17.5 psi |
| Initial oven temperature | 37°C | Split ratio | 70:1 |
| Initial time | 4.0 minutes | Split flow | 76.0 mL/min. |
| Oven ramp rate | 2°C/mm | GC outlet | MSD |
| Oven final first ramp | 60°C | Outlet pressure | Vacuum |
| Final time first ramp | 0 minutes | MSD transfer line temp. | 280°C |
| Oven ramp rate | 5°C/mm | Ion source temperature | 230°C |
| Oven final temperature | 280°C | Quadrupole temp. | 150°C |
| Final time | 20 minutes | MSD EM offset | 250 V |
| Total run time | 75.5 minutes | MSD solvent delay | 2.0 minutes |
| Inlet temperature | 280°C | MSD acquisition mode | TIC |
| Inlet mode | Split | Mass range | 29-550 a.u. |
| Carrier gas | Helium |  |  |

Supplementary Table 4: Reports of acetoin, acetyl propionyl or diacetyl in e-cigarette aerosols

| **Reference** | **Experimental** | **Puff Regime** | **DA** | **AP** | **Acetoin** | **Power** | **Comments** |
| --- | --- | --- | --- | --- | --- | --- | --- |
| Margham et al 2016 | Aerosol from 1 commercial product. 2 sets of 100 puffs | vol 55 ml, duration 3 sec, interval 30 sec | NQ <2.9 ng/puff in aerosol. | Not measured | Not measured | 4.6 Watts (2.85 ohms, 3.6 V) | Liquid concs not reported |
| Sleiman et al 2016 | Aerosol from 3 flavoured e-liquids. 1-5 puffs collected, initially or after steady state. 3.8 or 4.8 V, single or dual coil e-cigarettes | vol 50 ml duration 5 sec, interval 30 sec | Range 0.07-0.438 µg/mg liquid consumed, or 0.574-3.074 µg/puff. Single coil at 3.8V gave 2200 ng/puff. Liquid not analysed for DA | Not measured | Not measured | 5.6 watts (single coil EGO 2.6 ohm, 3.8 V) | Also identified DA in aerosols from neat VG & PG |
| Allen et al 2016 | Aerosols from 51 commercial e-liquids & e-cigarettes. | vol: undefined duration:8 sec interval: 15-30 sec | DA quantified in 38/51 aerosols. Range: 0.3-238.9 µg/cig. Liquids not analysed for DA. | AP quantified in 21/51 aerosols. Range 0.2-64.4 µg/cig. Liquids not analysed for AP. | Acetoin quantified in 48/51 aerosols. Range 1.3-529.2 µg/cig. Liquids not analysed for acetoin. | Various brands. Power not specified. | Smoking continued until liquids exhausted. No per puff yields reported. |
| Klager et al 2017 | Aerosols from 26 flavour/e-cigarette combinations. Puff volumes 45-80 mL at 1 min intervals. | vol: 45-80 mL duration:undefined interval: 60 sec | DA above LOD for 16/26 samples. Range 0.028-3.69 µg/m³ corresponding to max of 0.295 ng/80 mL puff | AP above LOD for 5/26 samples. Max aerosol concentration was 1.14 µg/m³ corresponding to 0.091 ng/80 mL puff | Acetoin above LOD for 17/26 samples. Max aerosol conc was 23.8 µg/m³ = 1.90 ng/80 mL puff | Various brands. Power not specified | Puff flow rates were set to minimum to activate the ecigarette. Similar protocol to Allen et al 2017 |
| Moldoveanu et al 2017 | Aerosols from 12 commercial e-cigarettes. | vol 55 ml, duration 3 sec, interval 30 sec | Aerosol DA yields: average 8.2 ng/puff, range 0.34-39.4 ng/puff, for 12 devices. | Aerosol AP yields: average 4.0 ng/puff, range 0.23-20.0 ng/puff. | Not measured | Not reported |  |
| Melvin et al 2020 | Aerosols from 8 commercial cig-a-like e-cigarettes | vol 55 ml, duration 5 sec, interval 30 sec | Aerosol DA yields: average 44 ng/puff, range 32-195 ng/puff, for 8 devices. | Aerosol AP yields: average 23 ng/puff, range 13-33 ng/puff, for 2 devices. | Not measured | Not reported |  |
| Present study | Aerosols from experimental e-liquids containing DA, AP | vol 80 ml, duration 3 sec, interval 30 sec | 2450 ng/puff from 1114 µg/mL DA in liquid. 28.8 ng/puff from 10 µg/mL DA in e-liquid | 1580 ng/puff at 522 µg/mL AP in liquid, 136 ng/puff at 60.5 µg/mL AP in liquid | 2810 ng/puff from 1169  µg/mL AC in liquid, 3390 from 760 µg/mL AC in liquid | 5 Watts |  |
|  | Thermal decomposition of VG | vol 80 ml, duration 3 sec, interval 30 sec | <LOD, then 54-369 ng/puff between 26.5-35W | <LOD, then 45-88 ng/puff at 32 and 35W | <LOD then 84 ng/puff at 35 W | 10-35 watts |  |
|  | Thermal decomposition of 1,2 PG | vol 80 ml, duration 3 sec, interval 30 sec | < LOD and 17.1-125 ng/puff at 18-35 W | <LOD or <LOQ | <LOD | 10-35 watts |  |
|  | Thermal decomposition of 1,3 PD | vol 80 ml, duration 3 sec, interval 30 sec | < LOD then 12-14 ng/puff at 30-35 W | 2.9-46 ng/puff at  32 - 35 W | <LOD | 10-35 watts |  |
|  | Thermal decomposition of sugars | vol 80 ml, duration 3 sec, interval 30 sec | 0.05% sucrose 15/37 ng/puff  7.5% sucrose 160/901 µg/puff | 0.05% sucrose <LOD/<LOQ  10% sucrose 18/33 ng/puff | <LOD | 10/20 watts |  |

N/A Not applicable

Supplementary Figure 1: Mass loss from the device (per collection of 25 puffs) as a function of device power setting for the PG-, 1,3-PD- and VG-containing e-liquids used in the study.


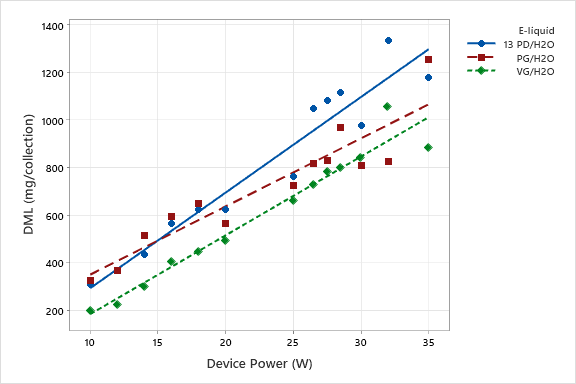


Supplementary Figure 2: The effect of different power settings on coil temperature for aqueous solutions of VG, PG and 1,3-PD


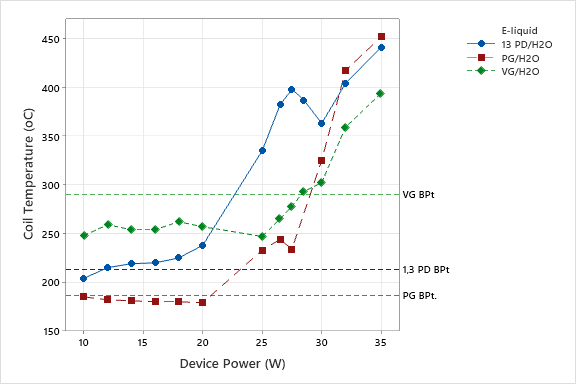


Supplementary Figure 3: Effect of temperature on AC production from the model e-liquids containing VG, PG and 1,3-PD


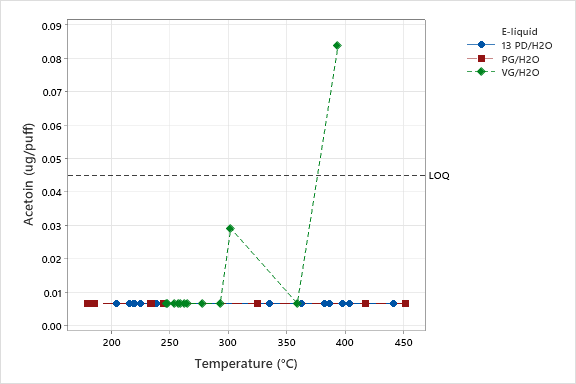


Supplementary Figure 4: Effect of temperature on AP production from the model e-liquids containing VG, PG and 1,3-PD


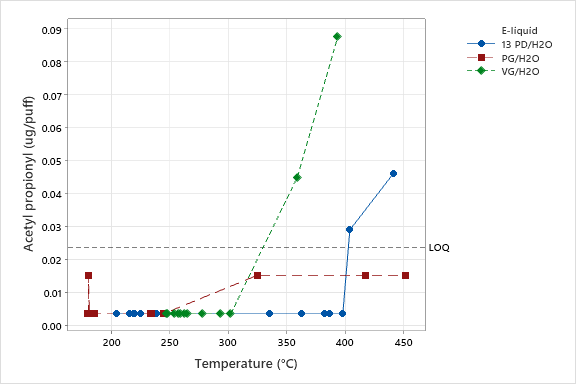

Supplement: Supplementary file 1 [file DataSheet1.docx]
